# mRNA vaccines expressing malaria transmission-blocking antigens Pfs25 and Pfs230D1 induce a functional immune response

**DOI:** 10.1038/s41541-023-00783-y

**Published:** 2024-01-06

**Authors:** Puthupparampil V. Scaria, Nicole Roth, Kim Schwendt, Olga V. Muratova, Nada Alani, Lynn E. Lambert, Emma K. Barnafo, Christopher G. Rowe, Irfan U. Zaidi, Kelly M. Rausch, David L. Narum, Benjamin Petsch, Patrick E. Duffy

**Affiliations:** 1 Laboratory of Malaria Immunology and Vaccinology, NIAID/NIH, Bethesda, 29 Lincoln Drive, Building 29B, MD 20892-2903 USA; 2 CureVac SE, Tübingen, Germany

**Keywords:** Malaria, RNA vaccines

## Abstract

Malaria transmission-blocking vaccines (TBV) are designed to inhibit the sexual stage development of the parasite in the mosquito host and can play a significant role in achieving the goal of malaria elimination. Preclinical and clinical studies using protein–protein conjugates of leading TBV antigens Pfs25 and Pfs230 domain 1 (Pfs230D1) have demonstrated the feasibility of TBV. Nevertheless, other promising vaccine platforms for TBV remain underexplored. The recent success of mRNA vaccines revealed the potential of this technology for infectious diseases. We explored the mRNA platform for TBV development. mRNA constructs of Pfs25 and Pfs230D1 variously incorporating signal peptides (SP), GPI anchor, and Trans Membrane (TM) domain were assessed in vitro for antigen expression, and selected constructs were evaluated in mice. Only mRNA constructs with GPI anchor or TM domain that resulted in high cell surface expression of the antigens yielded strong immune responses in mice. These mRNA constructs generated higher transmission-reducing functional activity versus the corresponding alum-adjuvanted protein-protein conjugates used as comparators. Pfs25 mRNA with GPI anchor or TM maintained >99% transmission reducing activity through 126 days, the duration of the study, demonstrating the potential of mRNA platform for TBV.

## Introduction

Malaria is a life-threatening disease that affects about half of the world’s population. WHO estimates approximately 247 million cases of Malaria worldwide in 2021, resulting in 619,000 deaths^1^. Preventive measures include insecticide-treated bed nets and intermittent presumptive treatments of children and pregnant women with drug combinations. These measures have contributed to substantial reductions in malaria burden during the past two decades, but progress has stalled and additional interventions such as vaccines are required to maintain control^1^.

Malaria vaccine development has been challenging in part due to the complex life cycle of the parasite spanning two different hosts, humans, and mosquitoes, and multiple stages of development such as sporozoite and liver stage, blood stage, and mosquito sexual and sporogonic stages^2–4^. Nevertheless, recent successes in vaccines against the liver-stage parasite have given hope that highly effective malaria vaccines can be developed^5–8^. RTS,S formulated in AS01E, the first licensed malaria vaccine, has an acceptable safety profile and reduced malaria episodes in 5–36 month-old children by 40% (*WHO malaria report 2021*). While this is highly encouraging, more effective vaccines will be necessary to control or eliminate malaria. Another sporozoite stage vaccine, R21 adjuvated with Matrix-M1, has shown promising efficacy in Phase 2 and Phase 3 trials^7,8^ and was approved by regulators for use in Ghana and Nigeria in 2023.

Transmission-blocking vaccines (TBV) targeting mosquito sexual stage antigens have garnered considerable interest recently to control parasite transmission from humans to mosquitos^9–11^. TBV does not prevent infection of the vaccine directly but rather offers protection to the community through herd immunity. TBV can be combined with vaccines targeting sporozoite, liver or blood stage to generate a multistage vaccine that may be more effective in controlling infection as well as onward transmission, thereby preventing the spread of escape mutants as well. Prominent candidate TBV antigens include Pfs25, Pfs230, Pfs48/45, and Pfs47^9,10^. These antigens, incorporated into various vaccine delivery platforms, have been evaluated extensively in preclinical studies for their transmission-blocking activity^12–22^. While early clinical studies focused on Pfs25 antigen, more recent studies also include Pfs230 and Pfs48/45 antigens [^23–27^, Clinical trial IDs: NCT02942277, NCT03917654, NCT05135273, NCT04862416, PACTR202201848463189, ISRCTN13649456]. The lead vaccine candidates in our laboratory are based on Pfs25 and Pfs230 domain 1 (Pfs230D1). Since these antigens are relatively small proteins with poor immunogenicity, we have developed a vaccine platform technology wherein the antigen is crosslinked with a carrier protein through chemical conjugation. Protein–protein conjugation results in crosslinked multimers with nanoparticle structures and significantly enhanced immunogenicity^12,17,28–30^. Pfs25 and Pfs230D1 candidates conjugated to carrier protein EPA (ExoProtein A) have advanced to clinical trials in malaria-naïve and malaria-experienced populations [^24–27^ NCT02942277, NCT03917654, NCT05135273].

mRNA vaccines have proven to be highly effective against SARS-CoV-2^31,32^. This has generated high levels of optimism that this technology could be effective against other pathogens^33–41^. The concept of expressing protein antigens for immune and therapeutic response by delivery of mRNA coding the protein has been known for over three decades^42–44^. While the inherent non-specific immune stimulatory properties of mRNA limited its applicability^42,45^, the demonstration that chemical modification of mRNA can modulate the inherent immune response detrimental to protein expression, paved the way to use this technology for vaccines^46,47^. Since then, there have been significant efforts by various companies to develop vaccines against different pathogens^31–41^. The success of mRNA vaccines against SARS-CoV-2 has validated the potential of this technology further and energized the field of mRNA vaccines^31,32^.

Currently, two different technologies are employed for mRNA vaccine development^48–52^. Licensed COVID-19 vaccines are based on the use of chemically modified nucleotides in the mRNA constructs to minimize the immunostimulatory response and thereby optimize tolerability and antigen expression^48–50^. This technology has also been used to evaluate mRNA for expression of Pfs25 and PfCSP in earlier studies^38,39^. These mRNAs were synthesized with m1 Ψ -5′-triphosphate instead of uridine 5′-triphosphate had 101 nucleotide-long poly(A) tails, and were capped with CleanCap. In this work, we employed sequence-optimized mRNA constructs composed of a 5′ cap structure, a GC-enriched open reading frame, a 3′ UTR, and a vector-encoded poly(A) stretch without any chemically modified nucleosides^51^. Both technologies use Lipid Nanoparticles for delivery of the mRNA payload in vivo and have been shown to yield antigen expression^52^. Here, we examined CureVac’s unmodified mRNA platform with two transmission-blocking antigens, Pfs25 and Pfs230D1, known to induce strong transmission-blocking activity in various preclinical and clinical studies of protein–protein conjugate candidates. We compared mRNA to protein-protein conjugate vaccines for immunogenicity.

As the cellular location of expressed antigens may impact the immune response^53,54^, we synthesized and screened several Pfs25 and Pfs230D1 mRNA constructs consisting of different signal peptides (SP), glycosylphosphatidylinositol (GPI) anchor and transmembrane (TM) domain in two different cell lines for antigen localization. Mouse immunogenicity studies showed mRNA constructs with GPI anchor or TM domain induced stronger immune response against the antigens indicating that cell surface presentation is critical for immune response against these two antigens. IgG subclass analysis revealed a Th1 bias for the immune response to mRNA candidates.

## Results

### Design and screening of mRNA constructs with various structural elements for expression

Intracellular delivery of mRNA has been shown to result in the expression of protein encoded by its sequence^43,44^. Immune responses induced against the expressed protein antigen may depend on its cellular location, which in turn is determined by elements present in the protein sequence. We constructed a series of mRNAs with different SP, GPI anchor, and TM domains (Tables 1 and 2) within the expressed ORFs to evaluate the impact of the cellular location of the expressed antigen and to maximize the immune response. Table 1 summarizes the cellular location and levels of Pfs25 when transfected with various mRNA constructs of Pfs25 (Supplementary Table 1 and Supplementary Fig. 1a–d). Cell surface localization was observed only when the GPI anchor or TM domain was present in the sequence, both showing similar levels of the antigen on the cell surface (Table 1: mRNAs A, D, E, I–K). These mRNA constructs also showed high levels of intracellular localization of the antigen by Western blot and FACS analyses. mRNAs with TM domain gave higher levels of protein in cell lysate compared to those with GPI anchor. Secreted antigen in the culture supernatant was only observed with mRNA that incorporated SP without GPI anchor or TM domain (Table 1: mRNAs F–H). These mRNAs also generated low levels of intracellular localization of the expressed antigen. Among the Pfs25 mRNAs with SP from Albumin or Insulin, the one with Albumin signal peptide generated the highest level of secreted antigen (Table 1: mRNA G).

**Table 1:** mRNA constructs of Pfs25 and localization of the expressed antigen at various cellular locations.

	Antigen–Pfs25		Antigen expression levels			
			Western (293 T)		FACS (HeLa)	
mRNA code	Pfs25 mRNA constructs tested for protein expression	Abbreviations of Pfs25 mRNAs selected for mouse immunogenicity study	Lysate	Supernatant	Intracellular	Surface
A	Pfs25 mRNA (+GPI anchor)		+	-	++	++
B	Pfs25 mRNA (sequence optimized for expression in Pichia)		-	-	-	-
C	Pfs25 mRNA (no SP; no GPI anchor)		-	-	-	-
D	Pfs25 mRNA (+Insulin SP; + GPI anchor)		+	-	++	++
E	Pfs25 mRNA (+Albumin SP; + GPI anchor)^a^	Pfs25 mRNA-GPI	+	-	++	++
F	Pfs25 mRNA (+Insulin SP)		+	+	+	-
G	Pfs25 mRNA (+ Albumin SP)^a^	Pfs25 mRNA-SP	+	++	+	-
H	Pfs25 mRNA (+Pfs25 SP)		+/-	+	+	-
I	Pfs25 mRNA (+ Pfs25 SP; + H1N1 TM)		++	-	++	++
J	Pfs25 mRNA (+ Insulin SP; + H1N1 TM)		++	-	++	++
K	Pfs25 mRNA (+ Albumin SP; + H1N1 TM)^a^	Pfs25 mRNA-TM	+++	-	++	++

*SP* signal peptide, *TM* transmembrane domain; - : not detected

^a^LNPs of mRNA constructs E, G and K were evaluated in a mouse immunogenicity study.

**Table 2:** mRNA constructs of Pfs230D1 and localization of the expressed antigen at various cellular locations.

	Antigen–Pfs230D1		Antigen expression levels			
			Western (293 T)		FACS (HeLa)	
mRNA code	Pfs230D1 mRNA constructs evaluated for protein expression	Abbreviations of Pfs230D1 mRNAs selected for mouse immunogenicity study	Lysate	Supernatant	Intracellular	Surface
A	Pfs230D1 mRNA (sequence optimized for expression in Pichia)		+	-	+/-	-
B	Pfs230D1 mRNA^a^	Pfs230D1 mRNA	+	-	+/-	-
C	Pfs230D1 mRNA (+Pfs230 SP)		-	-	-	-
D	Pfs230D1 mRNA (+Insulin SP)^a^	Pfs230D1 mRNA-SP	++	++	++	-
E	Pfs230D1 mRNA (+Albumin SP)		-	-	-	-
F	Pfs230D1 mRNA (+Insulin SP; +H1N1 TM)^a^	Pfs230D1 mRNA-TM	+++	-	++	++

*SP* signal peptide, *TM* transmembrane domain; - : not detected

^a^LNPs of mRNA constructs B, D and F were evaluated in a mouse immunogenicity study.

A similar analysis was done for different mRNA constructs of the Pfs230D1 antigen (Table 2, Supplementary Table 2, and Supplementary Fig. 2a–c). Among the six different constructs evaluated for Pfs230D1 antigen, only one construct with Insulin SP and TM domain generated a high level of antigen on the cell surface (Table 2: mRNA F). This construct also yielded high antigen levels in the intracellular compartment and cell lysate. Pfs230D1 with Insulin SP alone yielded antigen in the supernatant as well as an intracellular compartment (Table 2: mRNA D). Other constructs showed minimal or no antigen levels at different locations. (Data summarized in Tables 1 and 2 are presented in Supplementary Tables 1 and 2 and Supplementary Figs. 1 and 2)

### Evaluation of selected mRNA constructs in mouse immunogenicity study

Three mRNA constructs each for Pfs25 and Pfs230D1, indicated in Tables 1 and 2, were formulated as lipid nanoparticles (LNP/mRNA) and evaluated in mouse immunogenicity studies. mRNAs were selected based on the cellular location of the antigen to assess the effect of antigen location on immunogenicity. Of the mRNAs selected for Pfs25, two had strong cell surface expression as well as intracellular expression, but no antigen was detected in the supernatant (Pfs25 mRNA-GPI and Pfs25 mRNA-TM) (Table 1: mRNAs E and K respectively, Supplementary Fig. 1). Of these two, Pfs25 mRNA-TM had high levels of protein in the lysate. The third mRNA selected, Pfs25 mRNA-SP (Table 1: mRNA G), had a high level of protein in the supernatant and minimal or no expression at other locations (Table 1, Supplementary Fig. 1). Among the three mRNAs selected for Pfs230D1 (indicated in Table 2), Pfs230D1 mRNA-TM had high levels of antigen on cell surface, intracellular and cell lysate, but none in the supernatant (Table 2: mRNA F; Supplementary Fig. 2). Pfs230D1 mRNA-SP had high levels of antigen in cell lysate, supernatant and intracellular but none at cell surface (Table 2: mRNA D, Supplementary Fig. 2) whereas Pfs230D1 mRNA with no SP or TM had minimal levels in the lysate and no significant levels in other locations (Table 2: mRNA B, Supplementary Fig. 2).

The six selected mRNAs, indicated in Tables 1 and 2, were tested for immunogenicity in BALB/c mice. In addition, two groups of animals were immunized with combinations of Pfs25 and Pfs230D1 mRNAs to explore enhancement of serum functional activity or interference in the immune response. In combination-1, mRNAs of the two antigens with transmembrane domains that gave strong antigen expression at the cell surface were combined (Pfs230D1 mRNA-TM & Pfs25 mRNA-TM). Combination-2 consisted of Pfs230D1 and Pfs25 mRNAs with signal peptides that gave antigen expression in supernatant or intracellular locations but no cell surface expression (Pfs230D1 mRNA-SP and Pfs25 mRNA-SP). Combination groups received each antigen at the same dose as in their corresponding single antigen groups (5 µg of Pfs25 mRNA + 5 µg of Pfs230D1 mRNA).

### Antibody response in mice vaccinated with LNP/mRNA of Pfs25 and Pfs230D1

**Fig. 1:**
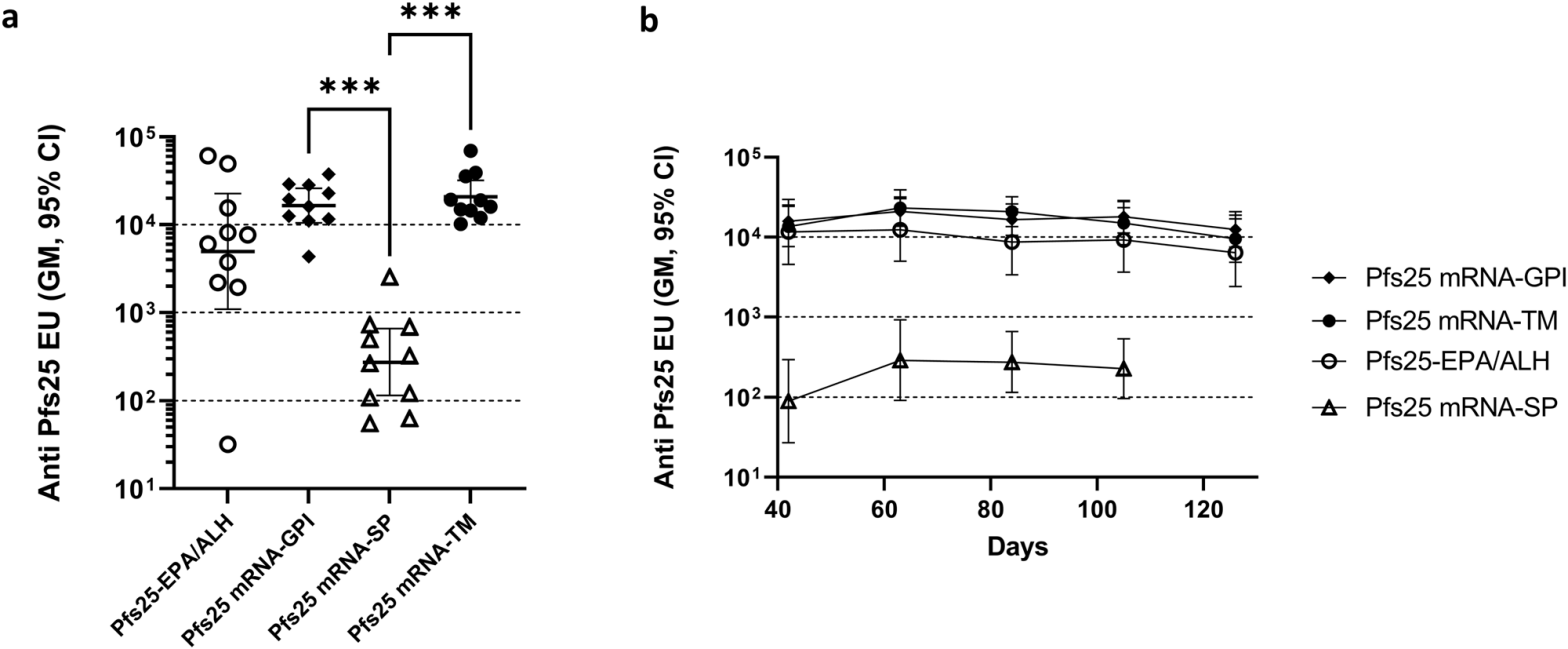
mRNAs encoding surface expressed Pfs25 antigen showed the highest levels of antibody response. **a** Anti-Pfs25 antibody levels in sera from mice vaccinated with Pfs25 mRNAs consisting of SP alone or SP with GPI anchor or TM, compared to Pfs25-EPA conjugate in Alhydrogel®. The figure shows the antibody levels on day 84 following vaccinations on days 0 and 21. **b** Serum antibody levels of all four immunogens at various time points from day 42 to day 126. Error bars represent the 95% confidence limit of the geometric mean. Statistical differences between groups were measured using a Kruskal–Wallis one-way ANOVA followed by a Dunn multiple comparator test. *p ≤ 0.05, **p ≤ 0.01, ***p ≤ 0.001, ****p ≤ 0.0001.

Immune sera obtained from mice were assayed for antibody responses against the two antigens, Pfs25 and Pfs230D1. Serum antibody responses to LNP/mRNA, were compared to those obtained from mice immunized with our benchmark EPA conjugates of the two antigens, formulated with Alhydrogel® adjuvant. Among the three Pfs25 mRNA constructs and the EPA conjugate studied, Pfs25 mRNA-GPI and Pfs25 mRNA-TM, mRNAs with GPI anchor or TM domain, induced the highest immune responses whereas the antibody response to the mRNA with SP alone, Pfs25 mRNA-SP, was moderate (Fig. 1a). Responses to mRNAs with GPI anchor or TM domain were significantly higher than that observed with mRNA incorporating SP alone. Antibody titer against the EPA conjugate was similar to that previously observed^12^ and was numerically but not significantly lower than the mRNA constructs with GPI or TM. Figure 1b shows the persistence of antibody levels over a period of 15 weeks following the second vaccination. Pfs25 mRNAs with GPI or TM, as well as the Alhydrogel® formulated Pfs25-EPA conjugate retained high antibody titer up to day 126 (Fig. 1b). The decrease in antibody titer from day 63, at peak titer, to day 126 was not significant for any of the three groups. Low antibody titers observed for Pfs25 mRNA with SP alone (Pfs25 mRNA-SP) also persisted without significant decrease.

**Fig. 2:**
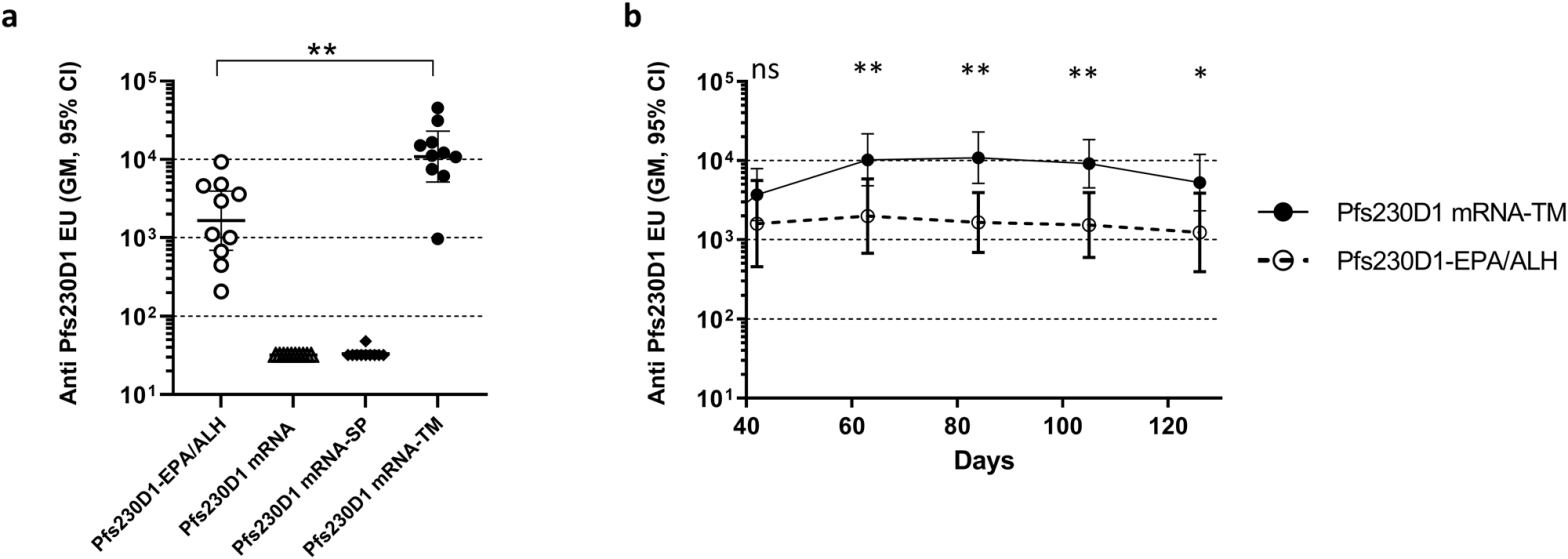
mRNA encoding surface expressed Pfs230D1 antigen showed the highest level of antibody response. **a** Anti-Pfs230D1 antibody levels in sera from mice vaccinated with Pfs230D1 mRNAs with or without SP or with SP and TM, compared to Pfs230D1-EPA conjugate in Alhydrogel®. The figure shows the antibody levels on day 84 following vaccinations on days 0 and 21. **b** Serum antibody levels of all four immunogens at various time points from day 42 to day 126. Error bars represent the 95% confidence limit of the geometric mean. Statistical differences between groups at each time point were measured using a Kruskal–Wallis one-way ANOVA followed by a Dunn multiple comparator test for (**a**) and a Mann–Whitney test for (**b**). *p ≤ 0.05, **p ≤ 0.01, ***p ≤ 0.001, ****p ≤ 0.0001.

Among the three Pfs230D1 mRNA groups evaluated in mice, only Pfs230D1 mRNA-TM that incorporates the TM domain, induced a strong immune response (Fig. 2a). Antibody responses generated by this mRNA increased from day 42 to day 63, and remained stable from days 63 to 105, and trended lower thereafter though the antibody titer on day 126 was not significantly different than that of day 63 (Fig. 2b). Antibody titer observed for this group was significantly higher than that of EPA conjugate of Pfs230D1 in Alhydrogel® at various time points tested during the study, except on day 42. Neither Pfs230D1 mRNA nor Pfs230D1 mRNA-SP showed any Pfs230D1-specific antibody response above background levels.

**Fig. 3:**
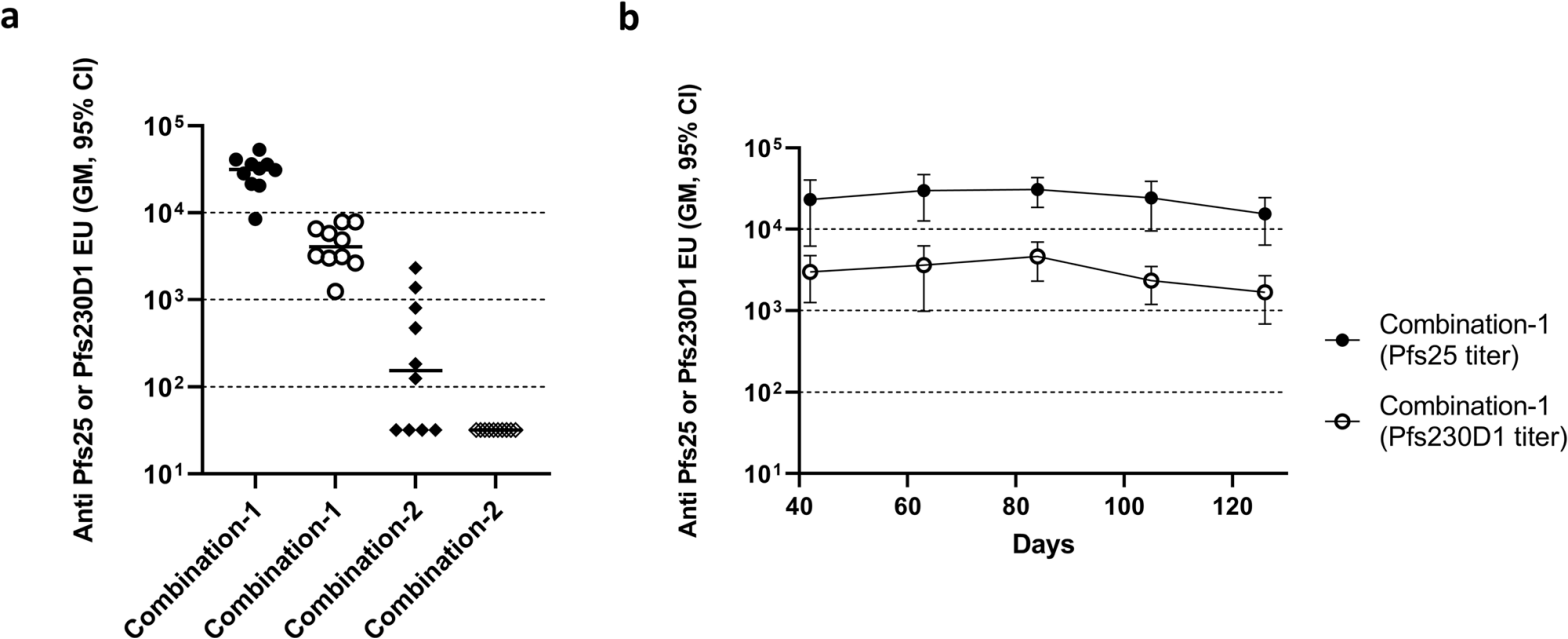
Combinations of Pfs25 and Pfs230D1 mRNAs encoding surface expression of antigens showed high levels of antibody against the two antigens. **a** Serum antibody levels, measured on day 84, of mice vaccinated with two combinations of Pfs25 and Pfs230D1 mRNAs. Combination 1 consisted of mRNA of the two antigens with SP and TM, and Combination 2 consisted of mRNAs of the antigens with SP alone. Closed symbols represent Pfs25 antibody titers, and open symbols represent Pfs230D1 antibody titers for the two combinations. **b** Serum antibody levels against Pfs25 and Pfs230D1 of Combination 1 at various time points from day 42 to day 126. Error bars represent the 95% confidence limit of the geometric mean.

While Combination-1 consisting of Pfs25 mRNA-TM and Pfs230D1 mRNA-TM, yielded high antibody titers against Pfs25 and Pfs230D1, Combination-2 consisting of mRNAs with SP alone (Pfs25 mRNA-SP + Pfs230D1 mRNA-SP) showed low levels of antibody response (Fig. 3a). This is consistent with responses observed for individual antigens Pfs25 and Pfs230D1, where mRNA constructs with TM induced high levels of antibody against these antigens whereas constructs with SP alone were poorly immunogenic (Figs. 1, 2). Antibody responses against both antigens, induced by Combination-1, persisted until day 126 without significant decline (Fig. 3b), as observed for individual mRNA constructs of Pfs25 and Pfs230D1 with TM, shown in Figs. 1, 2 respectively. Although the antibody response against Pfs25 mRNA-TM in combination-1 was similar to that observed for individual antigens (Supplementary Fig. 3a), Pfs230D1 antibody levels were significantly lower than those for the individual antigens (Supplementary Fig. 3b).

### Functional activity of immune sera from mice vaccinated with Pfs230D1 and Pfs25 mRNAs

**Fig. 4:**
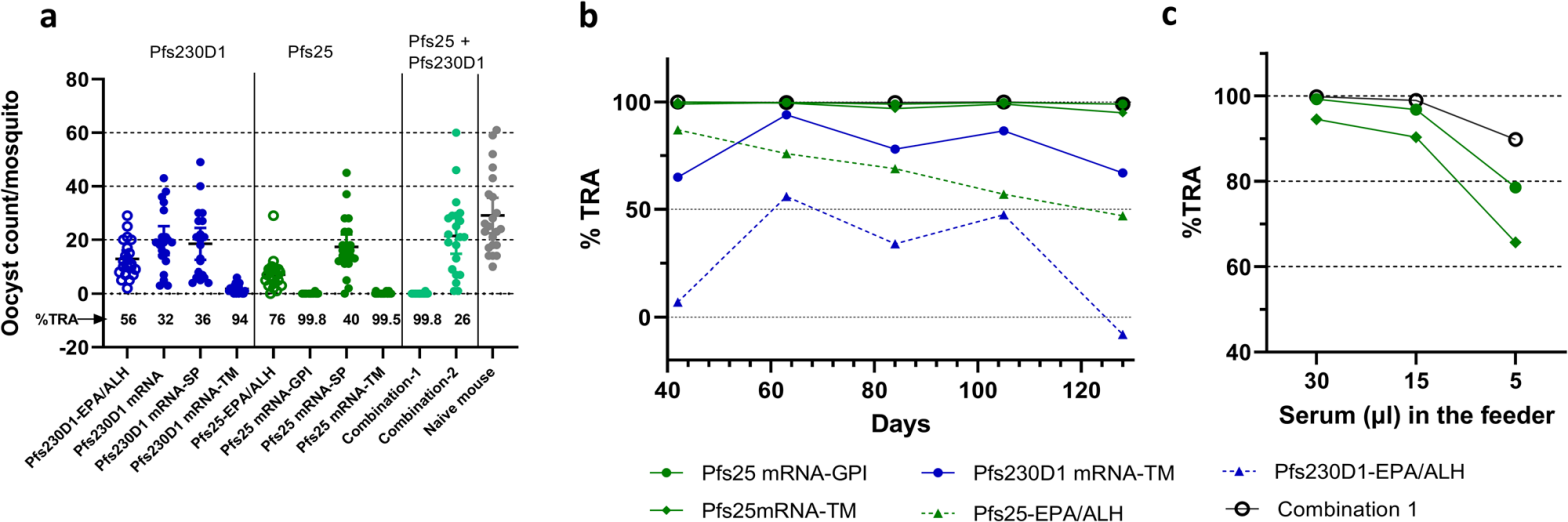
Vaccination with mRNAs encoding surface expressed antigens resulted in sera with high levels of transmission reducing functional activity. **a** Reduction in the midgut oocyst count of mosquitos fed on cultured *P. falciparum* parasites combined with the immune sera from mice vaccinated with the Pfs25 or Pfs230D1 mRNAs or EPA conjugates. SMFA was performed using sera collected on day 63, and mosquitos fed on test sera were compared with those fed on control sera from naïve mice to estimate the % reduction (% TRA). In total, 30 µl of pooled sera were diluted to 160 µl in the feeder. Error bars represent the 95% confidence limit of the geometric mean. **b** Transmission reducing activity of sera from mice that received various mRNAs and EPA conjugates of Pfs25 and Pfs230D1 at different time points from day 42 to day 126. **c** % TRA of day-126 sera from groups that received Pfs25 mRNA-GPI, Pfs25 mRNA-TM, and Combination-1, assayed using different dilutions (30, 15, or 5 µl diluted to 160 µl) of sera in the feeder.

Transmission-reducing activity (TRA) of mRNA expressed Pfs230D1 and Pfs25 antigens was assessed by Standard Membrane Feeding Assay (SMFA). Pfs230D1 mRNA-TM gave a TRA of 94% on day 63 (Fig. 4a). In contrast, Pfs230D1 mRNA and Pfs230D1 mRNA-SP gave low TRAs, 32 and 36%, respectively, consistent with a lack of significant antibody response by these constructs. Pfs230D1-EPA conjugate in Alhydrogel® gave a TRA of 56%. Pfs25 mRNA-GPI and Pfs25 mRNA-TM gave high levels of TRA, 99.8 and 99.5% respectively. Here again, the TRA observed for the mRNA with SP alone was low (40%). Pfs25-EPA in Alhydrogel® gave a TRA of 76%. Among the two combination groups, Combination-1 consisting of mRNAs with TM domain, gave high TRA (99.8%), whereas the combination-2 of mRNAs with SP did not show significant TRA (26%).

Pfs25 mRNA with GPI anchor or TM domain and the combination-1 retained high (>99%) TRA throughout the course of the study until day 126 (Fig. 4b). During this time, TRA for Pfs25-EPA conjugate decreased with time and had <50% TRA on day 126. Pfs230D1 mRNA-TM showed an increase in TRA from day 42 to day 63 but showed a decrease thereafter. Nevertheless, TRA remained >75% until day 105 and decreased below that level on day 126. TRA of Pfs230D1-EPA in Alhydrogel® was low during the study period and lost all the functional activity by day 126.

Since Pfs25 mRNA-GPI, Pfs25 mRNA-TM and Combination-1 retained >99% TRA till the end of the study, the d126 sera of these groups were re-analyzed at higher serum dilutions (5.3, 10.7, and 32-fold dilutions) to determine any difference in TRA between these groups (Fig. 4c). Comparing Pfs25 mRNAs with GPI anchor or TM domain, mRNA with GPI anchor showed higher TRA. Nevertheless, Combination-1 of Pfs25 and Pfs230D1 mRNA with TM gave the highest activity, ~80% TRA at 32-fold serum dilution.

### IgG subclass analysis of immune sera

**Fig. 5:**
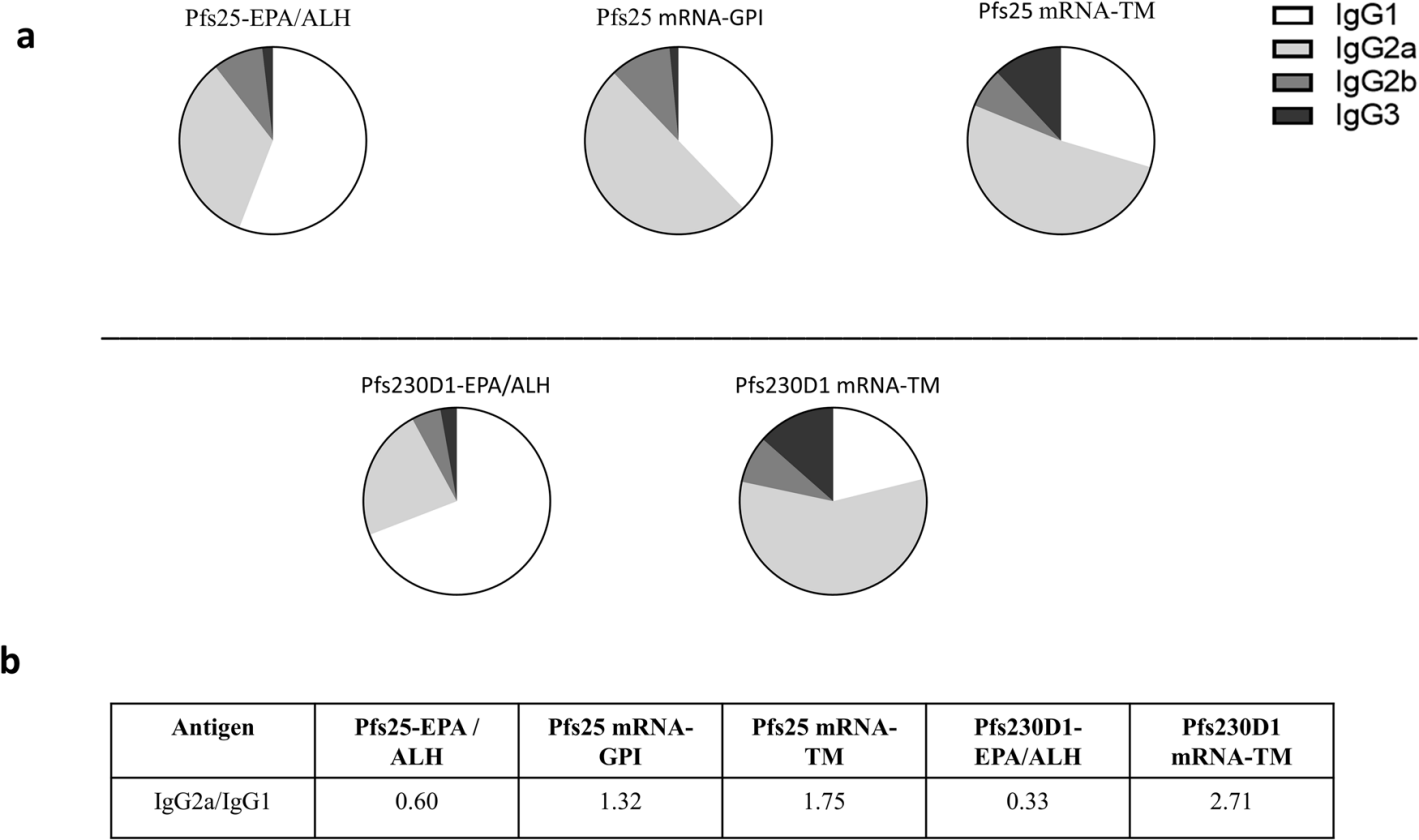
mRNA vaccines yielded a Th1-biased immune response. IgG subclass (IgG1, IgG2a, IgG2b, and IgG3) distribution of sera from mice vaccinated with **a** mRNAs of Pfs25 consisting of GPI or TM or Alhydrogel®-formulated Pfs25-EPA (Upper panel) and mRNAs of Pfs230D1 consisting of TM or Alhydrogel®0-formulated Pfs230D1-EPA (Lower panel). Immune sera collected on day 63 after two vaccinations (days 0 and 28) were analyzed by ELISA using pooled samples for each group using mouse monoclonal antibody isotype controls from Southern Biotech, Birmingham AL. **b** Ratio of IgG2a/IgG1 for various immunogens.

IgG subclass distributions of immune sera from day 63 for different groups with significant antibody titers were analyzed by ELISA. Pooled sera from various groups were assayed for IgG1, IgG2a, IgG2b, and IgG3. Sera from animals that received Pfs25-EPA in Alhydrogel® showed higher levels of IgG1 compared to other subtypes with an IgG2a/IgG1 ratio of 0.6 (Fig. 5a). In contrast, sera from Pfs25 mRNA groups were dominated by IgG2a. For Pfs25 mRNA groups, IgG2a/IgG1 ratios were 1.32 and 1.75, respectively, for constructs with GPI anchor and TM domain (Fig. 5b). A similar trend was observed in sera from animals that received Pfs230D1. In Alhydrogel®, Pfs230D1-EPA conjugate gave an IgG1-dominated response with an IgG2a/IgG1 ratio of 0.33, whereas IgG2a dominated the subclass distribution in the group that received Pfs230D1 mRNA-TM (IgG2a/IgG1 ratio of 2.71). These data indicate a Th-1-biased immune response to vaccination with mRNA constructs as opposed to a Th-2 response with protein conjugates in Alhydrogel®.

## Discussion

The success of the mRNA platform in generating effective SARS-CoV-2 vaccines has renewed interest in using this technology for vaccines against other infectious diseases. Once the target antigen is identified, mRNA-based vaccines can be generated rapidly by in vitro transcription from a plasmid encoding the antigen, followed by purification steps. Efficiency of antigen expression by mRNA depends on mRNA stability and its delivery into cells. Current mRNA vaccines use either nucleoside modification or sequence optimization to achieve mRNA stability and optimal antigen expression, as well as encapsulation by ionizable lipids for delivery of mRNA into cells. mRNA-expressed antigens may be targeted to different locations within the cell or secreted out of the cells based on the presence of elements such as SP, GPI anchor, TM domain, etc., incorporated into the mRNA sequence. The location of the expressed antigen may have a significant impact on the induction of immune response though this has not been explored adequately. In this study, we explored the effect of cellular location of antigen expression on the induction of immune response against two TBV antigens, Pfs25 and Pfs230D1.

We generated a series of mRNA constructs that directed two antigens to various cellular compartments such as cell surface, intracellular, and supernatant by incorporating combinations of SP, GPI anchor, or TM domain in their sequences (Tables 1 and 2). In vitro expression studies showed expression of these antigens at various cellular locations and the distribution of antigens depended on the targeting elements present in the mRNA constructs (Supplementary Figs. 1 and 2). The presence of GPI anchor or TM domain in combination with SP gave strong cell surface expression, as expected. In the absence of a GPI anchor or TM domain, antigens were located within an intracellular compartment or secreted into supernatant for constructs encoding a signal peptide fused to the antigen.

Mouse immunogenicity studies clearly showed that mRNAs with GPI anchor or TM domain were most effective in inducing an immune response. This was found to be true for both Pfs25 and Pfs230D1 antigens. mRNA constructs that yielded antigens localized in the supernatant or intracellular compartments generated limited or no antibody response after vaccination. These data clearly show that cell surface presentation may be required for efficient immune response, and mRNA designs should aim to achieve this.

Although we did not further elucidate the immunological mechanism behind this effect it seems reasonable to assume that presence on the cell surface will allow B cells to directly recognize the antigens, compared to intracellular expression. While secreted antigens are also accessible to B cells, we did not observe a similar positive effect on immunogenicity in our studies. Since this difference was not further investigated, we could not conclude if the amount of secreted vs. cell surface retained protein is identical or if total expressed protein amounts differ between the two constructs. Future investigations may examine if this effect could be mediated by protein design approaches leading to nanoparticle formation of secreted proteins as described before^55^.

Pfs25 and Pfs230D1 are two of the leading transmission-blocking antigens that are being evaluated in clinical trials^24–27^ [NCT02942277, NCT03917654, NCT05135273]. Pfs25 mRNAs containing GPI anchor or TM domain generated strong antibody responses after two vaccinations. This antibody response was maintained without significant decline for 105 days after the second vaccination on day 21. Antibody responses to the two mRNA constructs did not significantly differ from that of the Pfs25-EPA conjugate in Alhydrogel® adjuvant. These two mRNA constructs gave high levels of TRA > 99% in the Standard Membrane Feeding Assay, and this high activity was retained to the end of the study on day 126 (Fig. 4). In contrast, the conjugate in Alhydrogel® showed a continuous decline in TRA over time from >87% TRA at day-42 to below 47% on day-126, even though antibody levels were similar to those induced by the mRNAs (Fig. 1). This suggests that the mRNA vaccine may generate antibodies with higher functional activity compared to protein conjugates in Alhydrogel®. Pfs230D1 mRNA-TM also showed significantly higher antibody titer than that observed for Pfs230D1-EPA conjugate in Alhydrogel® and this was maintained for the duration of the study. TRA of Pfs230D1 mRNA-TM was lower than that of Pfs25 mRNA with TM or GPI at all time points and ranged between 84% and 65%. Nevertheless, TRA was higher than that of Pfs230D1-EPA conjugate in Alhydrogel® at all time points.

In a previous study, Hayashi et al.^39^ evaluated Pfs25 mRNA synthesized with chemically modified nucleosides. This mRNA induced high antibody levels and TRA (>94% TRA) 4 weeks after the third vaccination. In the current study, Pfs25 mRNAs with GPI or TM induced a prolonged antibody response against the antigen and serum functional activity (>99% TRA) lasting 15 weeks following two vaccinations. Even though the chemically modified mRNA induced higher antibody levels (anti-Pfs25 titers ~10^7^) compared to unmodified mRNA after two vaccinations (anti-Pfs25 titers ~10^4^), chemically modified mRNA did not yield a correspondingly higher level of functional activity. While comparisons between the two different mRNA types using results from different laboratories may be imperfect, it points to the need for a side-by-side comparison.

Combinations of multiple antigens have been pursued for increasing and broadening vaccine efficacy against the targeted pathogen. We evaluated whether mRNA combinations of two TBV antigens can generate higher functional activity compared to individual antigens. Among the two combinations tested, a combination of Pfs25 mRNA-TM and Pfs230D1 mRNA-TM (Combination-1) gave high functional activity (>99% TRA). Combination groups received each antigen at the same dose as the single antigen groups. Although Pfs230D1 mRNA-TM showed lower TRA compared to Combination-1, Pfs25 mRNA-TM alone showed high functional activity similar to Combination-1 (Fig. 4b). On further analysis at higher serum dilutions, Combination-1 yielded greater TRA compared to Pfs25 mRNA-TM alone. Huang et al.^56^ examined the immune response of recombinant Pfs25 and Pfs230C1 (an antigen with a similar sequence to Pfs230D1) incorporated on the surface of a liposome (CoPoP) individually and in combination in mouse and rabbit immunogenicity studies. While the total protein dose was the same for all the groups, this dose was divided between the two antigens for the combination group. In mice, the combination showed lower TRA compared to Pfs25 alone, whereas in rabbits, the TRA of the combination was similar to that of Pfs25, indicating that the combination does not provide additional benefit over a single antigen. A similar conclusion was reached in a study that compared viral vector expressed Pfs25, Pfs230C, and a fusion protein of the two antigens, where functional activity did not increase with the fusion protein^57^. In contrast, an increase in transmission-blocking activity was reported with a chimera of Pfs230 and Pfs48/45 functional domains compared to single antigens^58^. In our mRNA study, the Pfs25 and Pfs230D1 combination generated greater functional activity revealed at higher serum dilutions. At the highest serum dilution, Pfs25 mRNA-TM had a TRA of 66% while Combination-1 gave 90% TRA, suggesting further studies at different doses may be warranted to examine the benefit of antigen combination. Nevertheless, the increase in functional activity will need to be weighed against the increased cost and complexity of a combination vaccine.

While the primary goals of this study were to explore mRNA vaccination to generate an immune response in mice resulting in transmission-reducing functional activity and to assess the impact of antigen location on the immune response, a comparison of the two antigens is possible from the data generated. Previous studies comparing Pfs25 and Pfs230D1 antigens using their respective EPA conjugates have shown that Pfs230D1 may be a superior antigen compared to Pfs25 for transmission-blocking activity in humans but not mice^26^. Chemical conjugates of Pfs230D1 and Pfs25 with EPA in liposomal adjuvant containing TLR4 agonist and QS21 showed superior TRA activity for Pfs230 conjugate than the Pfs25 conjugate when tested in non-human primates^16^. A similar observation was made when these conjugates were tested in humans with Alhydrogel® as an adjuvant, even though mouse studies did not show a significant difference in their immunogenicity and TRA activity^26^.

Transmission blocking activity of Pfs25 and Pfs230 antigens is known to be antibody-mediated and to target functional epitopes^59–61^. The functional activity of the Pfs230D1 antibody is mediated by complement-dependent lysis of gametes^26,59,62^. Superior functional activity of the Pfs230D1 antigen observed in non-human primates and humans may be attributable to complement-mediated enhancement of the functional activity. Potential boosting of the immune response against this gamete antigen in malaria-exposed populations might also enhance or sustain functional antibodies. In this study, the first to directly compare the two antigens by mRNA platform delivery, the mRNA construct of Pfs25 appears to induce higher immunogenicity and serum functional activity compared to Pfs230D1 mRNA in mice. Intracellular expression of the antigen by mRNA may be expected to generate qualitatively different immune responses compared to extracellular antigen delivery of protein subunit antigens.

IgG subclass analysis showed a Th-1 biased immune response with higher IgG2a/IgG1 ratios from the mRNAs of Pfs25 and Pfs230D1 as opposed to a Th-2 biased response from Alhydrogel® adjuvanted protein-protein conjugates. Th-1 response is especially beneficial for Pfs230D1 antigen as this induces antibody subclasses that fix complement and activate classical complement pathway more efficiently. Nevertheless, the lower functional activity of Pfs230D1 mRNA compared to Pfs25 mRNA observed here is unexpected. Since mouse serum lacks strong complement activity, we supplement it with human serum during the performance of SMFA. Despite that, the functional activity of Pfs230D1 may be underestimated due to the presence of inhibitors of the complement pathway in mouse serum^63^. This points to the need for further evaluation of these mRNA constructs in non-human primates and possibly in humans.

The mRNA constructs of Pfs230D1 may need further optimization to improve antigen expression. Notably, the mRNA designs of Pfs230D1 and Pfs25 are based on the first-generation sequence optimization technology employed at CureVac, and further improvement may be achieved by the second-generation technology developed more recently at CureVac^64^. Since currently licensed mRNA products are based on modified nucleosides and promise to induce lower reactogenicity in clinical settings, assessing them in the context of this project seems to be a reasonable next step. Nevertheless, this study demonstrates that the mRNA platform can induce immune responses with transmission-blocking activity, and further optimization may improve this activity.

## Materials and methods

### mRNA design and synthesis

The mRNA vaccines are based on CureVac’s RNActive® platform (claimed and described in, for example, patents WO2002098443 and WO2012019780) and include no chemically modified nucleosides. They are composed of a 5′ cap structure, a GC-enriched open reading frame, a 3′ UTR, and a vector-encoded poly(A) stretch. LNP encapsulation was performed with Acuitas LNP technology (Vancouver, Canada). LNPs used in this study are composed of ionizable amino lipids, phospholipids, cholesterol, and PEGylated lipids. The mRNA-encoded protein is based on the Pfs25 and Pfs230D1 proteins of *Plasmodium falciparum*. mRNAs consisting of SP sequences from different proteins (Insulin, Albumin, etc.) were included in the design with or without TM or GPI anchor. TM used in various constructs was from H1N1 HA protein fused to the antigens.

### Screening for antigen expression

mRNA constructs were evaluated for expression of Pfs25 and Pfs230D1 antigens in two different cell types, 293 T and HeLa cells. For detection of mRNA expression in cell culture by FACS, HeLa cells were seeded in 6-well plates at a density of 400,000 cells/well. Twenty-four later, cells were transfected with 2 µg of mRNA per well via Lipofection. For this, RNAs were complexed with Lipofectamine 2000 (Life Technologies) at a ratio of 1:1.5 and transfected into cells according to the manufacturer’s protocol. Protein expression was assessed 24 h post transfection. For FACS analysis, cells were fixed and analyzed with intact (surface staining) or permeabilized plasma membranes via treatment with Perm/Wash buffer (BD Biosciences, Cat. 554723). Pfs25 protein expression was assessed by staining with Rat anti-Pfs25 polyclonal IgG (LMIV, MV-1541) and Pfs230 protein expression was assessed by staining with Rat anti-Pfs230 polyclonal IgG (LMIV, MV-1760) at 1:500 dilutions, followed by goat anti-rat IgG FITC antibody (Sigma-Aldrich, Cat. F1763) at 1:100 dilution in a BD FACS Canto II cell analyzer and the FlowJo^TM^ 10 software.

For detection of mRNA expression in cell culture by western blotting, 293 T cells were seeded in 6-well plates at a density of 500,000 cells/well. Twenty-four hours later, cells were transfected with 2 µg of mRNA per well via Lipofection. For this, mRNAs were complexed with Lipofectamine 2000 (Life Technologies) at a ratio of 1:1.5 and transfected into cells according to the manufacturer’s protocol. Protein expression was assessed 24 h post transfection. For western blotting, supernatants were transferred into tubes, and cells were lysed in 1× Läemmli buffer, proteins were separated on 4–20% Mini-PROTEAN® TGX™ Precast Protein Gels (BioRad) and transferred to a nitrocellulose membrane (Odyssey nitrocellulose membrane 0.22 µm; Li-Cor, Cat 926-31092). Specific proteins were detected using Rat anti-Pfs25 polyclonal IgG (LMIV, MV-1541) and Rat anti-Pfs230 polyclonal IgG (LMIV, MV-1760) at 1:1000 dilutions, followed by goat anti-rat IgG IRDye® 800CW (Li-Cor, Cat. 926-32219; 1:5000 dilution). Protein detection and image processing were carried out in an Odyssey® CLx Imaging system and LI-COR’s Image Studio version 5.2.5 according to the manufacturer’s instructions.

### Immunogenicity studies

Mouse immunogenicity studies were performed under the guidelines and approval of the Institutional Animal Care and Use Committee (IACUC) at the National Institutes of Health. Immunogenicity of various mRNA/LNP formulations and conjugates were evaluated in 5–6 weeks naïve female BALB/c mice purchased from Charles River Laboratories and housed in an NIH facility. All mRNA formulations were stored at −80 °C prior to use, thawed not longer than 4 hours prior to vaccination, and maintained at 4 °C until vaccination. Groups of 10 mice were used for each test sample. Mice were vaccinated by intramuscular injection of 20 µl test samples containing 5 µg of mRNA or 0.5 µg of Alhydrogel® formulated conjugates (antigen dose equivalent) on days 0 and 21. Each mouse was vaccinated with a separate syringe preloaded with 20 µl of the test sample. Groups that received combinations of mRNA were vaccinated with 5 µg each of the mRNAs for a total of 10 µg mRNA for each mouse. No adverse reactions were observed in any of the animals post vaccination. Blood samples from animals at various time points, including days −7, 0, 18 h, 21, 42, 63, 84, and 105, were collected by bleeding from a mandibular vein. At the end of the study, on day 126, animals were anesthetized by IP administration of 0.05 mL of ketamine/xylazine (2:1), bled by cardiac puncture, and euthanized by cervical dislocation. Sera obtained were analyzed for anti-Pfs25 and anti-Pfs230D1 antibody titer by ELISA and functional activity by standard membrane feeding assay (SMFA).

### Antibody levels and functional activity (ELISA and SMFA)

Antibody levels induced by vaccination and the functional activity of immune sera were assayed as described earlier^17^. Briefly, anti-Pfs25 and anti-Pfs230D1 antibody titers were assayed using ELISA with Pfs25 and Pfs230D1, respectively, as the plate antigens, as described earlier^17^. The functional activity of immune sera for various test groups was assayed using pooled sera from each group by Standard Membrane Feeding Assay (SMFA) that determines the TRA^17^. A set of *Anopheles* mosquitoes (20–25) were fed on test or control sera mixed with cultured *P. falciparum* gametocytes through a membrane-feeding apparatus. After 8 days, each mosquito was dissected to count the number of oocysts developed in the midgut. The ability of immune sera to block parasite development within the mosquito midgut was measured as the reduction in the number of oocysts present in the midgut of mosquitos fed on cultured gametocytes mixed with immune sera versus naive mouse control sera. TRA is defined as the percentage decrease in mean oocyst counts in mosquitos fed on immune sera compared to those fed on control sera.

### IgG subclass analyses

IgG subclass distribution of immune sera was analyzed by ELISA using pooled sera for each group. IgG1, IgG2a, IgG2b and IgG3 were assayed using mouse monoclonal antibody isotype controls from Sothern Biotech, Birmingham AL, (Cat. # 1073-04, 1083-04, 1093-04 and 1103-04 respectively for IgG1, IgG2a, IgG2b and IgG3) at 1:3000 dilutions. This was followed by incubation with an alkaline phosphatase labeled goat anti-mouse IgG (H + L) from KPL (Cat # 5220-0357) at 1:3000 dilution and treatment with alkaline phosphatase substrate to determine the ELISA titers for each IgG subclass. Data are expressed as % contribution to the sum of the subclasses^17^.

### Statistical analysis

ELISA and SMFA data were analyzed with Prism software (GraphPad Software, Inc., La Jolla, CA). Statistical differences between test groups (*P* ≤ 0.05) were measured using a Kruskal–Wallis analysis followed by a Dunn multiple comparator test for comparing three or more groups.

### Supplementary information

Supplementary Material

## Data Availability

The datasets generated during and/or analyzed during the current study are available from the corresponding author upon reasonable request.
