# Supplementary material for: mRNA vaccines expressing malaria transmission-blocking antigens Pfs25 and Pfs230D1 induce a functional immune response
Source: NPJ Vaccines. 2024 Jan 6;9:9. doi: 10.1038/s41541-023-00783-y (PMC10771442; doi:10.1038/s41541-023-00783-y)

Supplementary Table 1: List of various Pfs25 mRNAs with different targeting elements such as signal peptide, transmembrane domain, and GPI anchor, evaluated for in vitro antigen expression in 293T and HeLa cells.

	**Antigen – Pfs25**		**Antigen expression levels**			
			**Western (293T)**		**FACS (HeLa)**	
**mRNA Code**	**Pfs25 mRNA constructs tested for protein expression**	**Molecular weight (kDa)**	**Lysate**	**Supernatant**	**Intracellular**	**Surface**
A	Pfs25 mRNA (+GPI anchor)	~24	+	-	++	++
B	Pfs25 mRNA (sequence optimized for expression in Pichia)	~19	-	-	-	-
C	Pfs25 mRNA (no SP; no GPI anchor)	~19	-	-	-	-
D	Pfs25 mRNA (+Insulin SP; + GPI anchor)	~24	+	-	++	++
E	Pfs25 mRNA (+Albumin SP; + GPI anchor)**	~24	+	-	++	++
F	Pfs25 mRNA (+Insulin SP)	~21	+	+	+	-
G	Pfs25 mRNA (+ Albumin SP)**	~21	+	++	+	-
H	Pfs25 mRNA (+Pfs25 SP)	~21	+/-	+	+	-
I	Pfs25 mRNA (+ Pfs25 SP; + H1N1 TM)	~26	++	-	++	++
J	Pfs25 mRNA (+ Insulin SP; + H1N1 TM)	~26	++	-	++	++
K	Pfs25 mRNA (+ Albumin SP; + H1N1 TM)**	~26	+++	-	++	++

**LNPs of highlighted mRNA constructs were evaluated in mouse immunogenicity study

Supplementary Table 2: List of various Pfs230D1 mRNAs with different targeting signal peptides and transmembrane domain, evaluated for in vitro antigen expression in 293T and HeLa cells.

	**Antigen – Pfs230D1**		**Antigen expression levels**			
			**Western (293T)**		**FACS (HeLa)**	
**mRNA - Code**	**Pfs230D1 mRNA constructs evaluated for protein expression**	**Molecular weight (kDa)**	**Lysate**	**Supernatant**	**Intracellular**	**Surface**
A	Pfs230D1 mRNA (sequence optimized for expression in Pichia)	~22	+	-	+/-	-
B	Pfs230D1 mRNA**	~22	+	-	+/-	-
C	Pfs230D1 mRNA (+Pfs230 SP)	~24	-	-	-	-
D	Pfs230D1 mRNA (+Insulin SP)**	~24	++	++	++	-
E	Pfs230D1 mRNA (+Albumin SP)	~24	-	-	-	-
F	Pfs230D1 mRNA (+Insulin SP; +H1N1 TM)**	~29	+++	-	++	++

**LNPs of highlighted mRNA constructs were evaluated in mouse immunogenicity study

Supplementary Figure 1: (a) & (b): FACS analyses showing intracellular (a) and cell surface (b) localization of expressed Pfs25 antigen from constructs consisting of various targeting elements. (c) & (d): Western blot analyses showing localization of Pfs25 antigen in cell lysate (c) and supernatant (d) after transfection with various mRNA constructs.

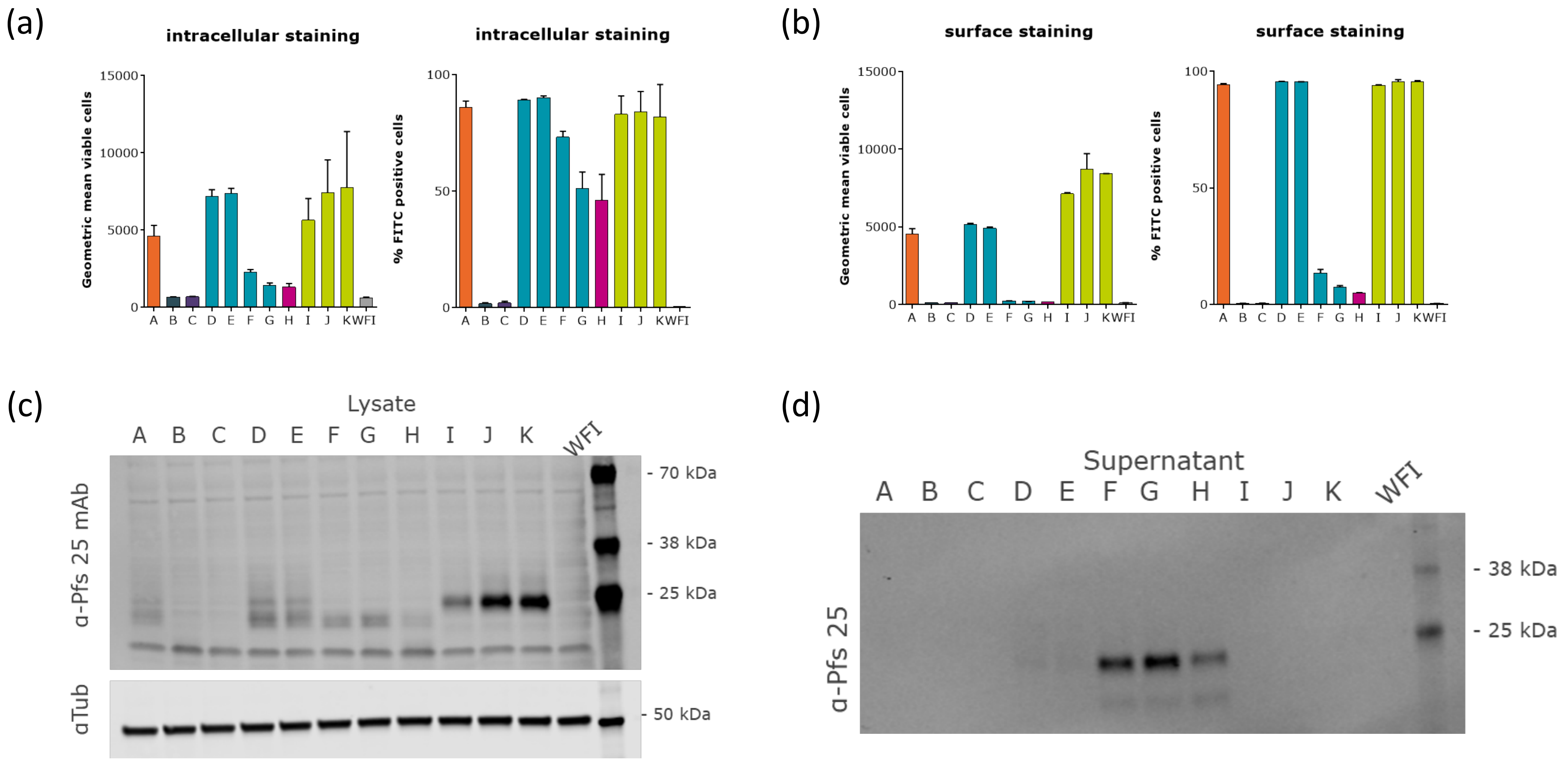

Supplementary Figure 2: (a) & (b): FACS analyses showing intracellular (a) and cell surface (b) localization of expressed Pfs230D1 antigen from constructs consisting of various targeting elements. (c): Western blot analyses showing localization of Pfs230D1 antigen in supernatant and cell lysate after transfection with various Pfs230D1 mRNAs.

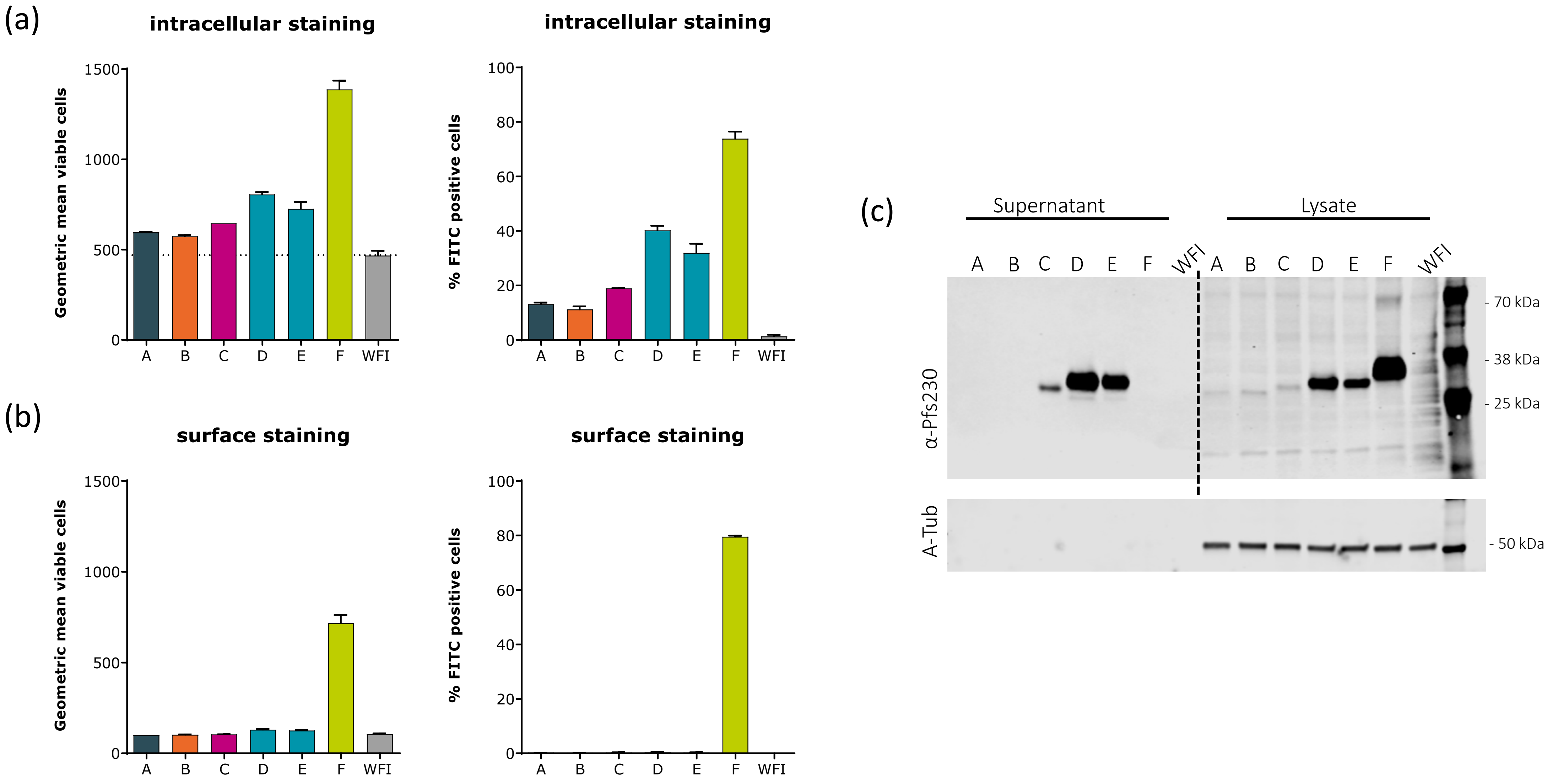

Supplementary Figure 3: Antibody response against Pfs230D1 in Combination-1 is significantly lower compared to same antigen administered alone. (a) Anti-Pfs230D1 antibody at various time points in mice immunized with Pfs230D1 mRNA with TM alone or in combination with Pfs25 mRNA with TM. (b) Anti-Pfs25 antibody at various time points in mice immunized with Pfs25 mRNA with TM alone or in combination with Pfs230D1 mRNA with TM. Statistical differences between groups were measured using Mann-Whitney test. *p≤0.05, **p≤0.01.

(a)

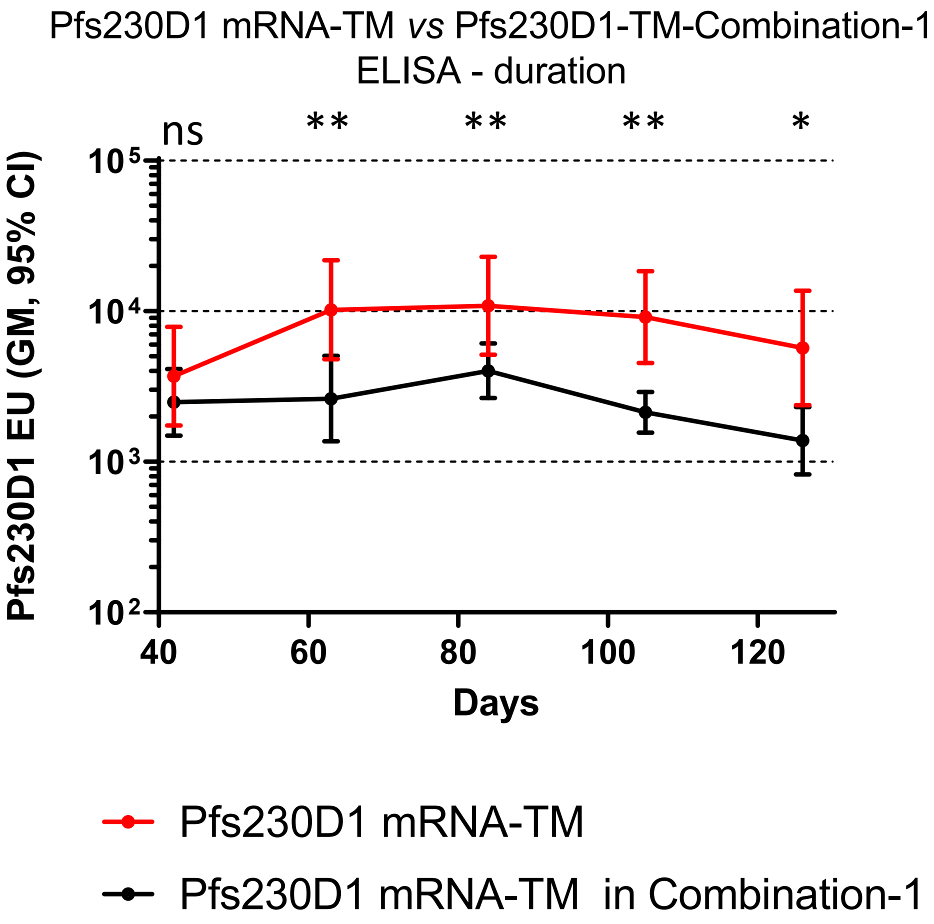

(b)

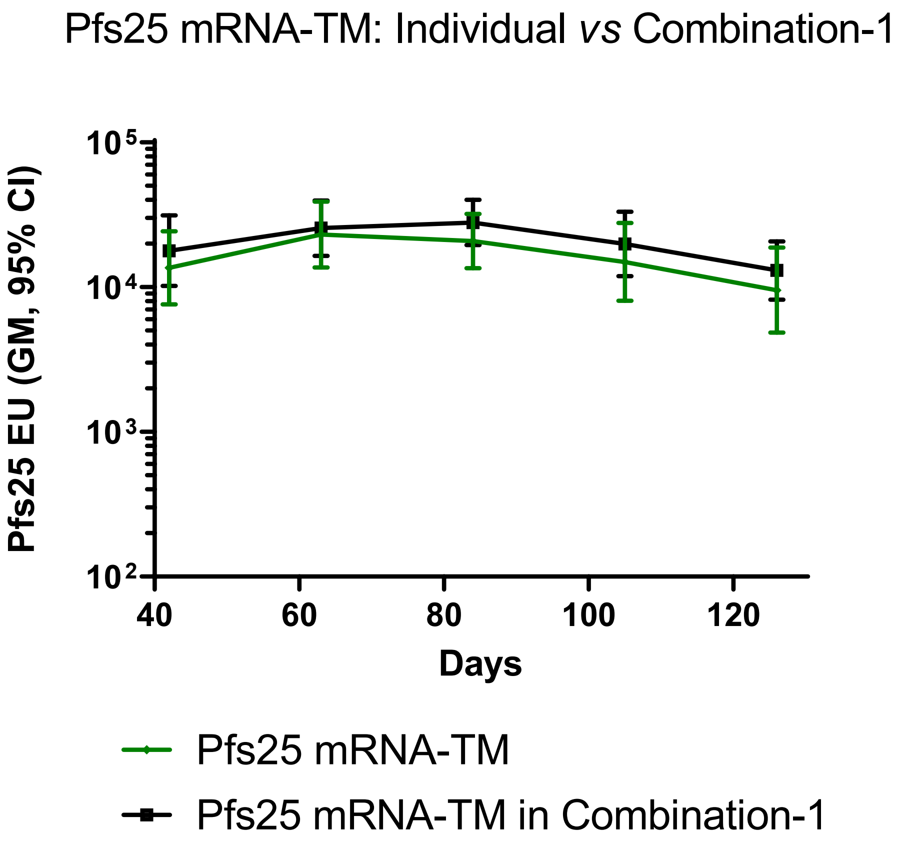

Supplement: Supplementary file 1 — Supplementary Material [file 41541_2023_783_MOESM1_ESM.pdf]
